# Yankee Brass

**Published:** 1883-09

**Authors:** 


### YANKEE BRASS.

We are asked through a very polite, but cool, note to give one of our handsomest notices to a brand new medical college, just inaugurated in a large city north of Mason and Dixon. But the graceful secretary forgot to inclose an *open sessime* in the form of a check, under the misapprehension, we suppose, that *cheek* is as good in Atlanta as *check*. "We didn't tumble to the suggestion," to use the expressive language of a *Constitution* reporter.

